# Effective Regulation of Gut Microbiota With Probiotics and Prebiotics May Prevent or Alleviate COVID-19 Through the Gut-Lung Axis

**DOI:** 10.3389/fphar.2022.895193

**Published:** 2022-04-25

**Authors:** Lei Xu, Chung S. Yang, Yanan Liu, Xin Zhang

**Affiliations:** ^1^ Department of Food Science and Engineering, Ningbo University, Ningbo, China; ^2^ Department of Chemical Biology, Ernest Mario School of Pharmacy, Rutgers The State University of New Jersey, Piscataway, NJ, United States

**Keywords:** COVID-19, gut microbiota, probiotics, gut-lung axis, prebiotics

## Abstract

Coronavirus disease 2019 (COVID-19) can disrupt the gut microbiota balance, and patients usually have intestinal disorders. The intestine is the largest immune organ of the human body, and gut microbes can affect the immune function of the lungs through the gut-lung axis. Many lines of evidence support the role of beneficial bacteria in enhancing human immunity, preventing pathogen colonization, and thereby reducing the incidence and severity of infection. In this article, we review the possible approach of modulating microbiota to help prevent and treat respiratory tract infections, including COVID-19, and discuss the possibility of using probiotics and prebiotics for this purpose. We also discuss the mechanism by which intestinal micro-flora regulate immunity and the effects of probiotics on the intestinal micro-ecological balance. Based on this understanding, we propose the use of probiotics and prebiotics to modulate gut microbiota for the prevention or alleviation of COVID-19 through the gut-lung axis.

## Introduction

Coronavirus disease 2019 (COVID-19) continues to spread worldwide, seriously threatening human health and profoundly impacting the global economy. Through the tremendous efforts of scientists around the world, the vaccines are available now. But with the constant emergence of variants of SARS-CoV-2, there is still a long way to go for the prevention and control of COVID-19. Many patients with COVID-19 showed dysbiosis in the intestinal tract, with a decrease in beneficial bacteria such as *Bifidobacteria*, suggesting the need to evaluate the patients’ gastrointestinal function (Chen and Vitetta, 2021; Livanos et al., 2021). It is known that the dysbiosis of the human gut microbiota is associated with various health conditions, including respiratory tract infections (RTI) via the gut-lung axis (Li et al., 2020). Therefore, the use of nutritional support and probiotics are recommended in patients with COVID-19 to regulate the balance of intestinal microbiota and reduce the risk of secondary infection (Badi et al., 2021; Sajdel-Sulkowska, 2021).

The immune system depends on adequate diet and nutrition to prevent infection. For instance, adequate protein intake is essential for optimal antibody production (Verduci and Köglmeier, 2021). Insufficiencies of micronutrients such as vitamin D and vitamin C are associated with an increased risk of infection (Jabczyk et al., 2021). Many recent meta-analyses have found that there is a positive correlation between vitamin D deficiency and the severity of COVID-19, and vitamin D supplementation can be used to prevent or reduce the severity of the disease (Meltzer, et al., 2020; Hadizadeh, 2021; Ma, et al., 2021). Intestinal malnutrition affects the local mucosa and indirectly decreases the immune response on the surface of the lung mucosa, increasing susceptibility to systemic inflammation (Rytter et al., 2014). Therefore, improving the nutritional status of patients and enhancing the body’s immunity through modulating microbiota is of significance for the treatment of new coronavirus pneumonia. To date, several clinical trials have successfully managed COVID-19 with probiotics as adjunctive therapy (Baindara et al., 2021; Pawar et al., 2021; Wu et al., 2021). In this article, we discuss the possible use of probiotics and prebiotics in the prevention of viral infection. In addition, the mechanism of action of probiotics against COVID-19 is discussed from the perspective of the gut-lung axis.

## Mechanism of Interaction of Gut-Lung *Axis*

### Gut Microbiota

A person has about 104 microorganisms in the gut, including bacteria, fungi, and viruses (Barko et al., 2018). Intestinal micro-organisms play an important role in the digestion and absorption of nutrients and promote the establishment of the immune system (Kolodziejczyk et al., 2019). A host with no gut microbiome cannot develop a functioning immune system, and the intestinal flora plays an essential role in maintaining the health of the body (Lerner et al., 2017). The host depends on the intestinal microflora to produce different metabolites through anaerobic fermentation (Jang et al., 2020). The monolayer of epithelial cells in the mucosa allows the passage of microbial metabolites and this interaction between microbes and the host cells affects the immune response and disease development (Nasr et al., 2020).

The mammalian gastrointestinal tract is not only the largest food digestion, absorption and metabolism organ in the body, but also the largest immunity organ. There is a large and relatively stable microbial community in the mammalian intestine (Le et al., 2020). The intestinal microflora starts to establish in the early life and affect the physiological function of the host (Bernardeau et al., 2017). After the outbreak of COVID-19, it was observed that critical COVID-19 patients with intestinal flora disorders were more susceptible to secondary infection and death (Spagnolello et al., 2021). Whether manipulating intestinal microflora can prevent and alleviate COVID-19 is an important topic for investigation.

### Lung Microbiota

The respiratory tract microbiota, similar to the intestinal microbes, mainly include *Bacteroides*, *Firmicutes*, and *Proteobacteria*, but their relative abundance is much lower, and the species diversity is also lower (Magryś, 2021). The microbial flora in the lungs migrated from the oral cavity, and spread along the mucosa most commonly by micro-aspiration of stomach contents (Ryck et al., 2014). The number and composition of the microbiota are affected by mucus-ciliary clearance or host defense mechanisms, temperature, oxygen tension, pH, nutrient availability, inflammatory cell activation, and bacterial competition (Yang et al., 2019). Airway microbiota were altered in patients with burns and inhalation injury, and the abundance of *Streptococcaceae* and *Enterobacteriaceae* increased by 30% in hypoxemia patients compared to patients with the partial pressure of arterial oxygen/fraction of inspired oxygen (PaO_2_/FiO_2_) ratio of more than 300; among the patients with PaO_2_/FiO_2_ ratio ≤300, *Prevotella melaninogenica* and *Corynebacterium* genus-level were significantly enriched (Walsh et al., 2017; Walton et al., 2021). Furthermore, PaO_2_/FiO_2_ < 274 mmHg is considered to be a reliable prognostic biomarker for COVID-19 patients (Sinatti et al., 2021). In a recent study in mice, it was found that after a respiratory infection, the relative abundance of *Bacteroides* increased and *Firmicutes* decreased (Groves et al., 2018). Changes in the lung microbiome may cause specific host immune responses and affect the prognosis of disease (Sommariva et al., 2020; Zhou et al., 2021).

The lung and airway microbiome directly affect immunity to disease and can change local immunity/inflammation during disease progression (Healy et al., 2021). RTI virus infection can directly cause immune damage to the respiratory tract and intestinal mucosa (Gautier et al., 2021). *Lactobacillus rhamnosus* GG (LGG) can maintain intestinal barrier homeostasis by enhancing intestinal mucin expression/barrier formation, reducing cell apoptosis and improving cell proliferation (Khailova et al., 2017). These may be the mechanisms for the protective effect of LGG on pneumonia in patients. Whether LGG has a protective effect on the intestines in COVID-19 patients remains to be determined.

### The Gut-Lung Axis

In recent years, hypotheses, such as “gut-liver” axis, “gut-brain” axis, “gut-heart” axis and “gut-kidney” axis, have been proposed (Galdeano et al., 2019). The “gut-lung” axis has been proposed to explain the relationship between intestinal flora and lung diseases (Standen et al., 2015). The intestinal micro-ecology can affect not only intestinal immunity but also extraintestinal immunity (Taghinezhad-S et al., 2021).

T-helper (Th)17 cells provide protection in barrier tissues but may also contribute to immune pathology (Bacher et al., 2019). Intestinal segmented filamentous bacteria can stimulate the body to produce Th17 immune cells and reduce the infection rate and mortality of *Streptococcus pneumonia* (Tanabe, 2013; Gauguet et al., 2015). In patients with respiratory disease, increased Th17 cytokine levels correlate with exaggerated inflammation and lung damage (Iwanaga and Kolls, 2019). Mice inoculated with *Lactobacillus johnsonii* can significantly reduce the lungs Th2 type inflammatory response (Segal et al., 2016), and *Bacteroides* can increase the number of regulatory T (Treg) cells and reduce the occurrence of inflammation (Samuelson et al., 2015). In addition, it has been reported that immunity cells can migrate through the blood to the lungs and intestines, forming another mechanism for the connection between the lungs and the intestines (Reibman et al., 2008).

Microbial invasion is the most common cause of RTI (Dickson et al., 2014). The respiratory tract has the physiological function of exchanging gas with the outside world and is exposed to the external environment. When microorganisms break through the respiratory defense, it would cause host respiratory infection (Turck et al., 2019). RTI can cause changes in the intestinal flora and intestinal functions (He et al., 2017). The intestine can alleviate the symptoms of RTI via recovering the microbiota, increasing the production of short-chain fatty acids (SCFAs), and improving the immunity (Liu et al., 2018). In addition to the main symptoms of cough, fever, chest tightness and fatigue, patients with COVID-19 (especially severe patients) may also have gastrointestinal symptoms (diarrhea, nausea, vomiting, etc.) (Yeoh et al., 2021). Therefore, a health gut micro-ecology plays an important role for COVID-19 prevention.

## The Relationship Between COVID-19 and Gut-Lung Axis

RTI is a disease with high morbidity and mortality (Wu et al., 2015). Studies have shown that lung damage during influenza virus infection is accompanied by gut damage, which is not directly caused by enteroviruses (Xu et al., 2021).

### Changes of Intestinal Microflora After Respiratory Virus Infection

The lung is an organ constantly exposed to micro-organisms through inhalation or subclinical micro-aspiration (Santacroce et al., 2020). Influenza infection can affect the composition of the intestinal flora, and the dysbiosis of intestinal flora may reduce the host’s antiviral immune response, thereby aggravating the lung damage caused by the infection (Marsland et al., 2015). For example, changes in the number of actinomycetes due to gut environment may aggravate the damage in inflammatory bowel disease (Lin et al., 2017). The gut microbiota plays a vital role in the response of the lung to bacterial infections (Sundararaman et al., 2020). *P. aeruginosa*, *K. pneumoniae*, and *S. pneumoniae* are associated with increased morbidity and mortality during the acute pulmonary infection (Brown et al., 2017). Gut-lung interaction affects the role of bacteria in respiratory diseases, and the immune dialogue is a two-way process (Darbandi et al., 2021).

In a recent cross-sectional prospective study on 30 COVID-19 patients with 10 healthy volunteers as a control group, it was found that the increase of intestinal *Proteobacteria* and the decrease of *Firmicute*, and these changes may have triggered an abnormal mucosal immune response (Khan et al., 2021). The imbalance of the intestinal microbiota is an important predisposing factor of the severity of COVID-19 disease and the depletion of *B. plebeians* and *F. prausnitzii* in the guts further aggravates the situation by causing cytokine overproduction and dysregulated inflammation (Khan et al., 2021). In addition, three bacterial members from the Firmicutes phylum, the genus *Coprobacillus*, the species *Clostridium ramosum* and *C. hathewayi*, were positively associated with COVID-19 disease severity (Zuo et al., 2020). In many COVID-19 patients, the abundance of fecal *Bacteroides dorei*, *Bacteroides thetaiotaomicron*, *Bacteroides massiliensis*, and *Bacteroides ovatus*, were inversely correlated with SARS-CoV-2 load in fecal samples in many patients (Zuo et al., 2021). These studies illustrate the intestinal microflora changes can be caused by respiratory virus infection, and some intestinal microflora may affect SARS-CoV-2 infection (Goossens et al., 2020).

**FIGURE 1:**
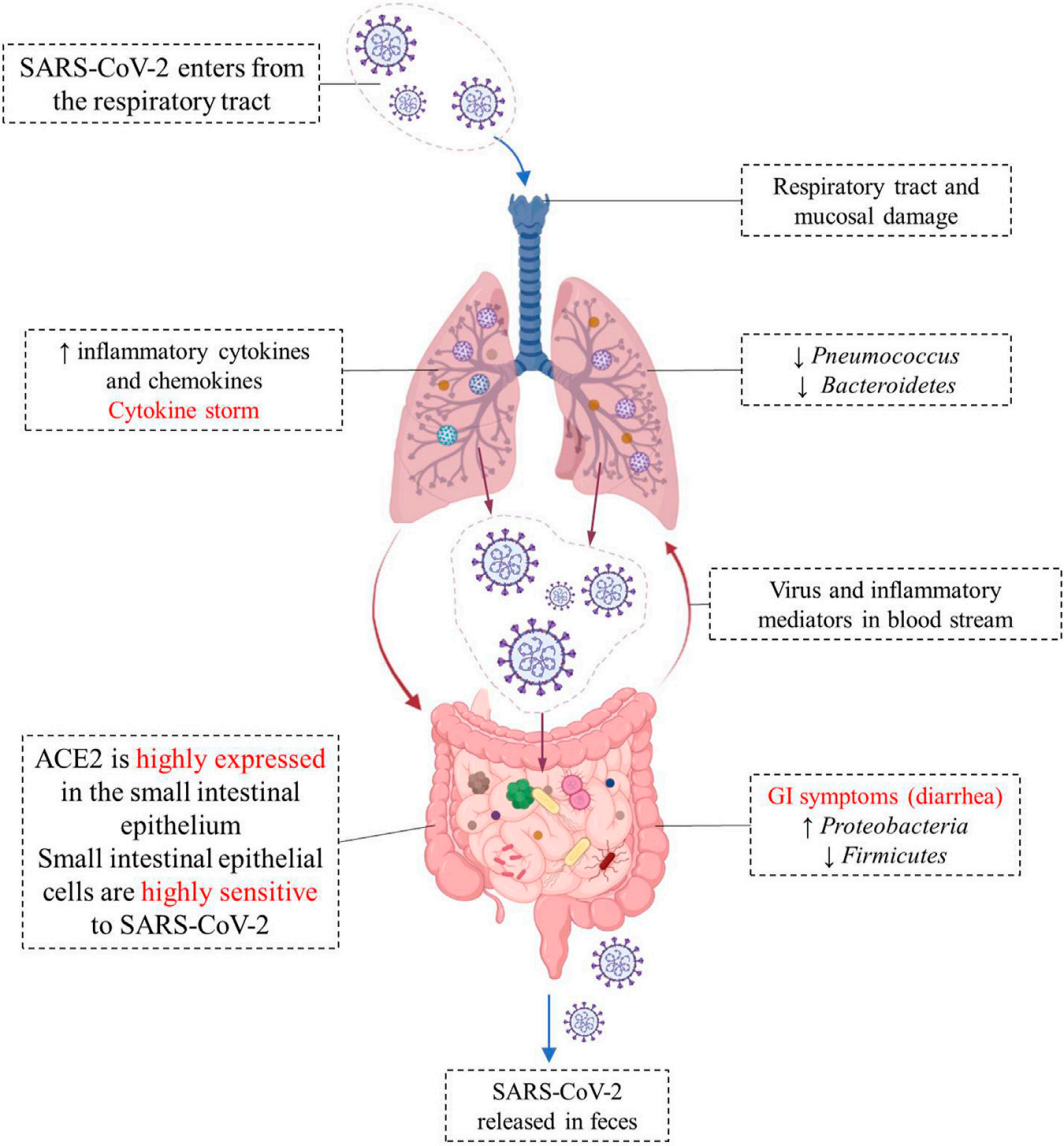
Different changes in the “gut-lung axis” after infection with COVID-19. Respiratory tract infection increases the production of cytokines and chemokines and causes changes in the microbial composition of microflora. Changes in the lung microbiome may cause specific host immune responses. The high expression of ACE2 in the small intestine makes small intestinal epithelial cells highly sensitive to SARS-CoV-2, and the viral infection leads to intestinal malabsorption, secretion imbalance, and diarrhea. SARS-CoV-2 RNA has been detected in fecal sample of COVID-19 patients.

SARS-COV-2 virus enters the cell using angiotensin-converting enzyme 2 (ACE2) as a receptor, which is abundantly expressed on the cell surface of glandular epithelial cilia and gastric and intestinal epithelial cells (Liang et al., 2020; Shang et al., 2020). The high expression of ACE2 in the epithelial cells of the small intestine makes them highly susceptible to SARS-CoV-2 infection (Yeung et al., 2012). SARS-CoV-2 may disrupt the function of ACE2, leading to intestinal malabsorption, secretion imbalance, stimulation of the enteric nervous system, and diarrhea (Wong et al., 2020). Meanwhile, cytokines are exaggeratedly released, promoting the recruitment of other cells, and leading to a huge inflammatory response (Lee et al., 2020). ACE2 also modulates innate immunity and influences the composition of the gut microbiota (Hashimoto et al., 2012; Penninger et al., 2021). Viral RNA was detected in fecal samples from 50% of COVID-19 patients, reflecting gastrointestinal infection (Cheung et al., 2020). The different changes of gut-lung axis after SARS-CoV-2 infection are illustrated in Figure 1.

### The Role of Gut-Lung Axis Microbiota in Immune System Regulation

Changes in the intestinal microbiome often alter in the respiratory tract immune response and homeostasis (Selwal et al., 2021). Microbes can regulate the lungs’ immune response by producing bacterial ligands and metabolites and affect the final composition of the lung flora (Budden et al., 2017). SCFAs-producing gut microbiota can deliver SCFAs to the lungs, thereby facilitating the generation of signals that initiate lung immunity (Liu et al., 2021). Specific gut microbes (such as *Staphylococcus*, *Streptococcus*, *Lactobacillus*, and *Bifidobacterium*) can affect respiratory diseases such as asthma, chronic obstructive pulmonary disease, and influenza virus infection (Chung, 2017). For example, *Lactobacillus paracasei* consumption can allow an early activation of pro-inflammatory cytokines (IL-1α, IL-1β) and a massive recruitment of immune cells in the lungs, mice fed *Lactobacillus* paracasei showed reduced susceptibility to influenza infection (Belkacem et al., 2017). Critically ill patients with COVID-19 disease can cause overall changes in the intestinal mucus layer (decreased thickness, reduced lumen coverage, poor adhesion) and intestinal barrier function (El-Sayed et al., 2021). Under the immunosuppressive environment, activation of the Th1 and Th17 cell immune responses by muramyl dipeptide and tuftsin fusion protein (MT) could result in the better suppression of Treg cells among CD4^+^T cells, Th17 cells generated following the MT induction enhanced the specific immune response at the mucosal surface (Jiang et al., 2014). Interleukin (IL)-17 plays an important role in mediating host defense against viruses and chronic lung disease (Gurczynski and Moore, 2018). IL-17 can lead to a significant increase in neutrophil and promote pulmonary fibrosis (Gasse et al., 2011). γδ T cells are tissue-resident cells that produce IL-17, and the microbiota-derived metabolites (particularly propionate) that inhibit intestinal γδ T cell production of IL-17 and IL-22 (Dupraz et al., 2021). In COVID-19 patients, the increase of IL-17 was positively correlated with the increase of lung injury, which suggested that IL-17 could be used as a biomarker of disease severity (Pacha et al., 2020). Therefore, targeting IL-17 is an effective strategy to prevent COVID-19 and alleviate the damage of SARS-CoV-2 in immunology.

The gut has long been thought to be the driving force behind multiple organ dysfunction syndromes (Klingensmith and Coopersmith, 2016). The balance of gut microbes plays a crucial role in the body’s immune system, and if this balance is upset, the immune system may collapse or spin out of control (Thirumdas et al., 2021). Intestinal microbiota disorders are associated with RTI, and the health of the microbiota is related to the conditions of other organs or tissues, including the lungs (Tao et al., 2020). SCFAs produced by the intestinal microbial fiber fermentation, support immune function by serving as signaling molecules on resident antigen-presenting cells to attenuate the inflammatory response (Wang et al., 2019). Intestinal microbes also induce immune tolerance by inhibiting unnecessary inflammatory reaction mechanisms through host adaptive immune evolution (Simčič et al., 2019; Hamzelou, 2020). Recent investigations showed that gut-mucosal immunity and gut flora play a role in the pathogenesis of HIV infection. Probiotics have been shown as a novel strategy to attenuate or prevent gastrointestinal involvement and to improve gut-mucosal immunity in HIV-infected subjects (Ceccarelli et al., 2019). A stable and nutritious intestinal micro-environment is a key factor in maintaining a healthy gut-lung axis. However, the blind use of conventional probiotics for COVID-19 is not recommended until we have further understanding of the pathogenesis of SARS-CoV-2 and its effect on gut microbiota, as the rationale for using probiotics in COVID-19 is derived from indirect evidence (Mak et al., 2020).

## Probiotics and COVID-19

The “gut-lung axis” concept can be explored for the prevention and treatment of COVID-19 with probiotics. Treatment of respiratory virus infection involves antagonism of respiratory viruses and regulation of cellular immunity and humoral immunity (Zelaya et al., 2020). In addition, probiotics may also be used to help regulate and maintain the balance of intestinal micro-ecology, thereby reducing the incidence of secondary bacterial infections.

### Application of Probiotics for the Prevention and Treatment of Respiratory Diseases

Beneficial bacteria interact with the mucosal surface to stimulate the immune response and reject pathogens from the intestinal epithelium through competitive rejection (Littman and Pamer, 2011). In addition, after oral administration of probiotics, the probiotics are believed to affect the relationship between commensal microorganisms and mucosal immunity, changing the basic and induced inflammatory balance in response to viral infections (Hardy et al., 2013). In a recent clinical trial of 70 COVID-19 patients, 42 received standard medical treatment, and 28 received additional oral beneficial bacterial treatment (including *Streptococcus thermophilus* DSM 32345, *Lactobacillus acidophilus* DSM 32241, *Lactobacillus brevis* DSM 27961, and other bacteria). The results showed that the estimated risk of respiratory failure in patients receiving oral bacterial treatment was 8 times lower. Among patients who did not receive oral bacterial treatment, the prevalence and mortality of patients transferred to the intensive care unit were higher (d'Ettorre et al., 2020).

In some COVID-19 patients, the intestinal *Bifidobacteria* and *Lactobacilli* were significantly reduced, this is could be an indicator of their weak immunity (Walton et al., 2021). In a meta-analysis of 20 randomized controlled trials, the effects of probiotics (especially *Lactobacillus* and *Bifidobacterium* strains) on the duration of acute RTI in otherwise healthy children and adults were analyzed. These results showed that the consumption of probiotics significantly reduced the duration of RTI disease onset, the number of days each person was ill, and the number of days absent/work/school. Children treated with probiotics had a lower risk of requiring antibiotic prescriptions than children who were not treated (King et al., 2014). In addition, on a total of 58 patients hospitalized with COVID-19, 24 received oral probiotic therapy (including *Bifidobacterium*, *Lactobacillus*, and *Streptococcus*) during hospitalization, while 34 received only standard care (no supplementation with oral bacteria). The results showed that patients taking probiotics had increased serum arginine, asparagine, and lactate levels, which could prevent the development of chronic fatigue by better-utilizing glucose and energy pathways (Santinelli et al., 2022). As secondary infections may be a serious issue in COVID-19, in addition to anti-inflammatory regimens, treatment with probiotics may be an adjuvant or alternative modality. However, the specific types and dosage of probiotics needs to be studied in future clinical trials.

### Probiotics Enhance Intestinal Immunity and Prevent Viral Immune Injury

Probiotics can be used to treat a variety of diseases, including viral infections (Kanauchi et al., 2018). The use of probiotics can also suppress inflammatory cytokines and help clear viral infections to minimize lung damage (Schiavi et al., 2016). Beneficial bacteria from probiotic supplements can stick to the surface of the epithelium, block the attachment of viruses by their spatial position, cover receptor sites in a nonspecific way, or compete for specific receptors (Etienne-Mesmin et al., 2019).

*Bifidobacteria* is mainly used to prevent and treat human intestinal diseases, because it can be colonized in the intestines (Salminen and Isolauri, 2006). Adhesion to the intestinal mucosa is the prerequisite for *Bifidobacteria* colonization and persistence in the gastrointestinal tract (Zhang et al., 2020). The attachment of *Bifidobacteria*, causes secretion of *Bifidobacteria* adhesin, which may bind to the receptor proteins of intestinal epithelial cells, and inhibit the colonization of pathogenic microorganisms (Westermann et al., 2016). A review of a total of 33 clinical trials related to viral respiratory infections found that beneficial bacteria from probiotic treatment also induced mucosal regeneration and intestinal mucins binding to viruses, lowered viral adhesion to epithelial cells and viral replication, and reduced the risk or duration of respiratory tract infection (Lehtoranta et al., 2014). In another study, among COVID-19 patients who received oral *Bifidobacteria* treatment, the interleukin-6 levels decreased and the mortality rate was decreased to 5% (from as 25%) (Bozkurt and Bilen, 2021). This approach should be further explored for treatment of hospitalized patients with moderate/severe COVID-19.

**FIGURE 2:**
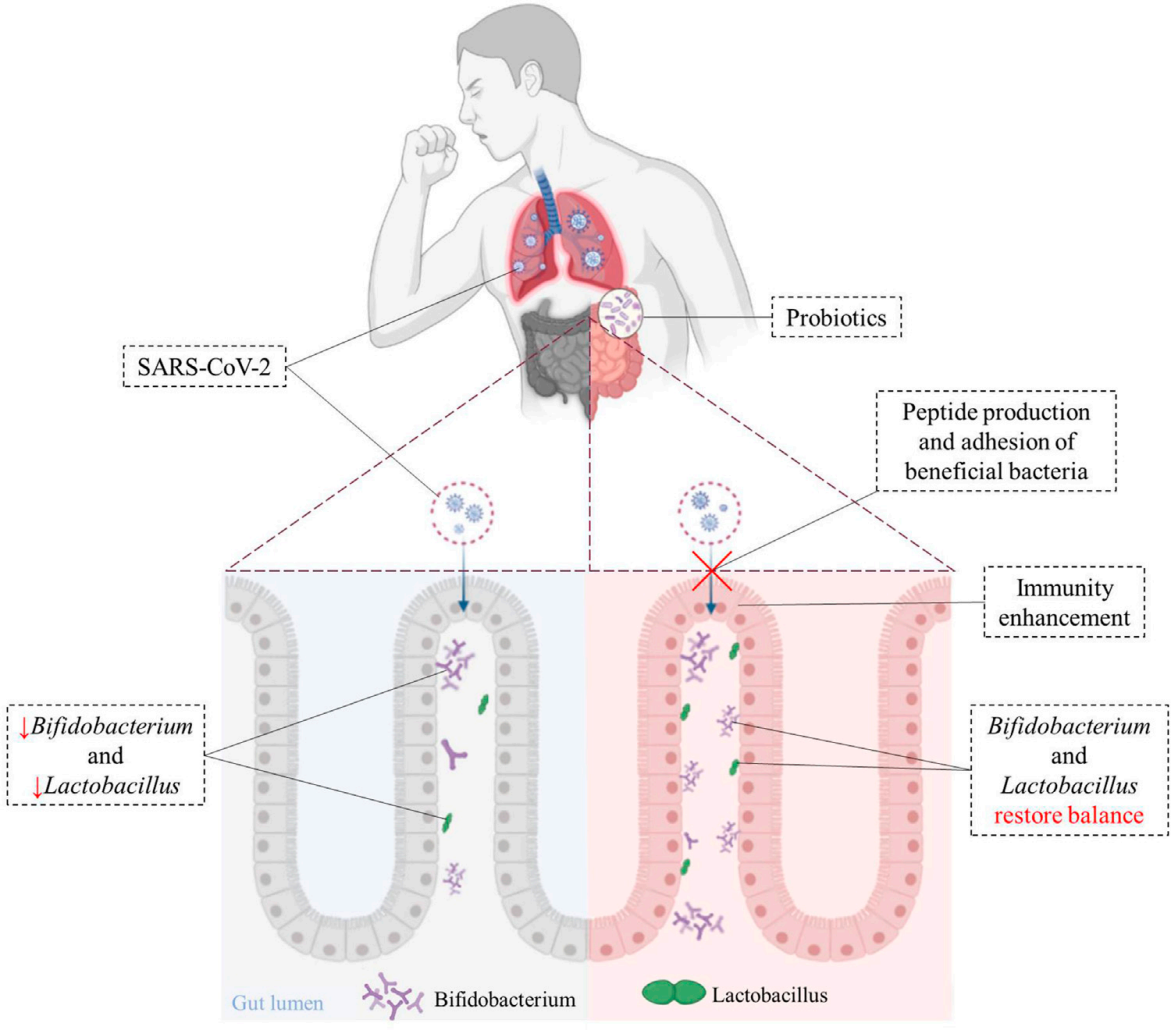
Immunoregulatory effects of probiotics on cells after SARS-CoV-2 infection. Beneficial bacteria from oral probiotics can block the binding of SARS-CoV-2 to the host cells, and helps improve gut dysbiosis caused by SARS-CoV-2 and hastens recovery in patients. The use of probiotics can suppress inflammatory-cytokines and help clear viral infections.

Peptides produced by some beneficial bacteria (such as *Lactobacillus* and *Paenibacillus*) have been shown to bind to ACE2 *in vitro*, and may block the binding of SARS-CoV-2 to target cells (Minato et al., 2020). Beneficial bacteria have also been shown to trigger antiviral immunity to promote the elimination of viruses (Din et al., 2021). Intranasal pre- and post-treatment of neonatal mice with LGG significantly improved mice survival after influenza virus infection (Kumova et al., 2019). LGG pretreatment was shown to improve immune gene transcriptional responses during early infection. Recent studies indicated that *Lactobacillus gassier* could be used as an auxiliary nutritional therapy in purine management, to reduce virus replication and help the treatment of COVID-19 (Morais et al., 2020). Figure 2 illustrates that beneficial bacteria originated from oral probiotics may block the binding of SARS-CoV-2 to the host cells, reduce virus replication, and increase the host’s immunity to the virus.

Overall, probiotics can improve the immune response to infections and this activity may also apply to SARS-CoV-2 infection. The effects of probiotics have a high degree of strain specificity. The transfer of sufficient numbers of bacteria to the effect site in the intestine is crucial for the successful treatment of COVID-19 patients. More laboratory and clinical studies are needed in this area.

### Proper Use of Probiotics

Although probiotics are known to be safety, they should be used with caution in patients with severely impaired immune function and premature infants (Sabina, 2014). LGG has been shown to cause bacteremia in some immunocompromised patients (Gupta and Garg, 2009). The dose-effect of *Lactobacillus Acidophilus* NCFM strain on the T cell immune response of rotavirus vaccination in a sterile pig model showed that probiotics may be ineffective or even harmful if not used in the proper dose (Wen et al., 2012). For patients critically ill, at risk of fungal infection or with central venous catheters the use of *Saccharomyces boulardii* should be avoided (Boyanova and Mitov, 2012). It has also been reported that LGG causes sepsis in cardiac surgery patients (Kochan et al., 2011).

The long-term effects of probiotic use are unknown and need to be studied in randomized controlled trials. Individualized and specific probiotic administration methods may be necessary, because empirical supplementation of probiotics may not produce uniform and lasting effects on the mucosa (Celiberto et al., 2018). Probiotic dietary supplements are usually made by preservation of bacterial strains, mainly through lyophilization or microencapsulation, and marketed in the form of tablets, capsules and freeze-dried formulations (Salehi et al., 2020). SARS-CoV-2 infected patients may present lesions in the lungs compromising their gas exchange capability. *In vitro* data suggested that the probiotic formulation SLAB51 can reduce nitric oxide synthesis in intestinal cells (Ceccarelli et al., 2021). Studies have shown that 10^8^–10^9^ CFU/g is an ideal probiotic dose for health benefits (Gurram et al., 2021). Certain strains of lactic acid bacteria modulate both innate and acquired immunity when administered at an adjusted dose of 10^9^ colony forming units (CFU)/day (Roobab et al., 2020). A study has proposed that the number of probiotics required must be consumed in a sufficient amount (>7 log CFU) to protect and treat respiratory infections, including COVID-19 (Olaimat et al., 2020). For patients with COVID-19, probiotics can be used to maintain the intestinal microecological balance and prevent secondary bacterial infections (Oliveira et al., 2021). More research is required into these types of bacteria to determine their effectiveness as probiotic formulations.

Probiotics can alter the composition of intestinal flora, improve the immune function, and subside intestinal inflammatory response (Plaza-Diaz et al., 2019). To date, no published studies have reported the use of probiotics as an add-on therapy for the treatment of COVID-19. Scientists and clinicians around the world are also studying the relationship between the gut microbiome and susceptibility to COVID-19 and evaluating the effects of various probiotic strains in reducing viral load through different mechanisms. The application of oral probiotics in the clinical treatment of COVID-19 still needs to be studied further in large-scale randomized clinical trials. In addition to clinical data, eating patterns, genetic associations, and environmental exposure should also be considered.

## Potential Application of Prebiotics for the Prevention of COVID-19

The definition of prebiotics has been revised several times. The International Scientific Association for Probiotics and Prebiotics (ISAPP) proposed in 2017 that the definition be revised to “a substrate that is selectively utilized by host microorganisms conferring a health benefit” (Gibson, et al., 2017). Prebiotics can alter the composition of the intestinal flora, and provide energy for the growth of beneficial bacteria (Calatayud et al., 2021). In addition, prebiotics can improve the function of the intestinal barrier and stimulate the production of metabolites beneficial to the host (Davani-Davari et al., 2019). Foods containing prebiotics, such as fiber, oligosaccharides, and polyphenols, can enhance the growth of beneficial bacteria (Parnell and Reimer, 2012; Lordan et al., 2020; Wiciński et al., 2020). For example, inulin-enriched diet stimulated the growth of *Bifidobacterium* and *Bacteroides* (Trompette et al., 2018); and whole grain cereals can modify bacterial profiles increasing relative quantities of *Bifidobacterium* and *Lactobacilli* (Tuohy et al., 2012). In principle, prebiotics should reduce the risk of a person to contract COVID-19 or alleviate the syndromes (Gasmi et al., 2020; Batista et al., 2021). However, more laboratory and human studies are needed to test this hypothesis.

Fermentable dietary fiber can promote the production of SCFAs (Koh et al., 2016), which enhance intestinal barrier function (Makki et al., 2018). Improving gut barrier function reduces the entry of harmful microorganisms and their metabolites into the host circulatory system (Knudsen et al., 2018; Loo et al., 2020). Fermentable dietary fiber has been shown to alter the number of gut bacteria in rodents and the concentration of SCFAs in their fecal (Cui et al., 2019). Dietary fermentable fiber and SCFAs could balance innate and adaptive immunity, thereby promoting resolution of influenza virus infection (Trompette et al., 2018). Mice fed a high-fiber diet and butyrate exhibited reduced neutrophil infiltration, enhanced T cell bioenergetics, relieved lung inflammation, and facilitated influenza virus clearance (Visan, 2018).

Oligosaccharides can fight against pathogenic viruses by the direct interaction with the virus (negative charge on the surface of viruses), inhibiting the invasion and adsorption of viruses, and also hindering the obstruction of the viral transcription and replication via direct interference (Iravani and Varma, 2021). A recent study investigated the antiviral effects of resveratrol oligosaccharides for SARS-CoV-2. Human MRC5 lung cells infected with SARS-CoV-2 were incubated with various concentrations of resveratrol oligosaccharides. This result indicated that resveratrol oligosaccharides at 5 and 10% effectively decreased the infection of the MRC5 cells by SARS-CoV-2 (Hamada et al., 2021). Dietary intervention with fructans and galactooligosaccharide (GOS), leads to a higher abundance of *Bifidobacterium* and *Lactobacillus* (So et al., 2018). GOS cannot be digested by humans but can be fermented by commensal bacteria in the intestines (Wilson and Whelan, 2017). GOS has shown to be a substrate of commensal bacteria *Lactobacilli* and increase the abundance of *Lactobacilli* and *Bifidobacteria* (Gonai et al., 2017). In a human study, three dose levels of fructo-oligosaccharides (FOS) (2.5, 5, and 10 g/day) or placebo (maltodextrin 10 g/day) were given to 80 participants. The results showed that the consumption of FOS increased the relative abundance of *Bifidobacteria* and *Lactobacilli* (Tandon et al., 2019). After 8 weeks of prebiotic (oligofructose) treatment to mice under physiological stress (high-fat diet), the modulation of the taxa and functional gut microbiota was found to be associated with the beneficial effects on obesity and related metabolic disorders such as a decrease in inflammation, a decrease in plasma leptin levels and an improvement of glucose homeostasis (Everard et al., 2014). In a recent randomized controlled trial on overweight or obese children, compared to children given placebo, those received prebiotic oligofructose-enriched inulin lost 3.1% of weight. The species of *Bifidobacterium* increased significantly, while the number of *Bacteroides* vulgaris decreased significantly (Nicolucci et al., 2017).

Polyphenols, found in many foods and beverages are known to affect gut microbiota in favor of the growth of beneficial bacteria (Jayabalan et al., 2014; Singh et al., 2019; Ma and Chen, 2020; Plamada and Vodnar, 2021). Many studies have shown tea polyphenols can improve the intestinal microecology by regulating the diversity and quantity of intestinal flora (Chen and Yang, 2019; Yan et al., 2020; Sun et al., 2021). Herein, some of these studies are used as examples to illustrate the prebiotic activities of polyphenols. (-)-Epigallocatechin gallate (EGCG), the most abundant and biologically active catechin in green tea, has been shown to significantly increase the production of SCFAs and the population of the *Bifidobacterium* (Zhang et al., 2013). Oral administration of green tea extract (equivalent to EGCG doses of 300–400 mg/kg) in drinking water to mice, almost completely prevented interstitial and peribronchial fibrosis (Donà et al., 2003). EGCG has shown anti-viral activities including the inhibition of SARS-CoV-2 infection (Ohgitani et al., 2021; Tallei et al., 2021). For example, following the infection of H9N2 swine influenza virus in mice, daily administration of EGCG (10 mg/kg, i. g.) for 5 days significantly prolongs mouse survival and reduces the death rate from 65 to 35% (Xu et al., 2017). With demonstrated activities in alleviating pulmonary fibrosis in rodent models and humans, EGCG and green tea extract may be useful for the prevention and treatment of pulmonary fibrosis in COVID-19 patients (Zhang et al., 2021).

Many epidemiological studies have shown that tea consumption is associated with lower incidence of obesity, metabolic syndrome, and related diseases (Yang and Zhang, 2019). Similar beneficial activity has also been shown for dietary fiber and oligosaccharides (Shoaib et al., 2016; Holscher, 2017; Myhrstad et al., 2020). High body mass index has been recognized as an important risk factor for COVID-19 developing into a critical state, especially abdominal obesity (Földi et al., 2021), and obesity is associated with an increase in Th17 and IL-17 (Tarantino et al., 2014). Therefore, weight loss and improved metabolic health may help better cope with COVID-19, whether regular drinking tea (and the required amount) can reduce the risk of COVID-19 infection and related syndromes needs to be further investigated.

## Conclusion

In this article, we illustrate the importance of gut microbiota in the body’s immune system and anti-viral functions. The gut microbiome also affects lung health through the gut-lung axis. Viral infection, such as SARS-CoV-2 infections in the lung, can also cause intestinal microbiota dysbiosis. Therefore, maintaining a healthy intestinal microbiota with a healthy diet is important. The use of probiotics and prebiotics to enhance the development of healthy microbiota is a promising approach to prevent and alleviate viral infection. This includes the prevention and treatment of COVID-19, to alleviate associated symptoms and prevent secondary infections. Some published successful examples and mechanisms of action in this area are discussed; however, more studies are needed to substantiate this approach. It is also important to determine the specific probiotics or prebiotics and the optimal dosage to be used for the purpose of prevention and for adjuvant therapy in COVID-19 patients. In a practical sense, studies on the effects of probiotics and prebiotics on common influenza virus caused respiratory diseases are easier and may have a broader application. Such studies may also help us to deal with COVID-19. During SARS-CoV-2 infection in the respiratory tract and gastrointestinal mucosa, many disorders occur. Detailed multi-omics studies may be needed to analyze the changes in the gut and lung during and after the infection.
